# Editorial

**Published:** 1875-06

**Authors:** 


### Editorial.

### SALICYLIC ACID.

This agent as a disinfectant and antiseptic is receiving considerable attention; and from the experience of those who have been testing it, it possesses a high degree of merit, and for certain purposes it would seem must supersede every thing else that has hitherto been used.

Those, both dentists and surgeons, who have been using it, speak in the highest terms of it. We are beginning to use it, and shall speak of it more fully when we experimentally know more about it. From the evidence which is contained in this number of the REGISTER, we can hardly entertain a doubt of its great value. We ask a careful reading of the articles on the subject.

### DENTAL EDUCATION.

By private communication we learn that an appropriation has been made to establish a dental department in Michigan University. This is a great step for the dental profession in Michigan, one which they will doubtless appreciate and foster. There is a large and flourishing medical college in connection with this Institution, and we see no reason why a dental college may not flourish as well.

This Institution is large, well established and largely endowed, and well prepared for carrying out any educational scheme it may set on foot. It is free from the embarassments and unfavorable influences of various kinds, that are usually attached to smaller and special enterprises. Then a high standard can be established, which it seems almost impossible to do in most of the existing dental colleges. It will make no competition with any other colleges, but will simply take its position and maintain it.

If it does its work better than others have done, it will be a matter of gratification not only for the good it will do, b utit will serve as a stimulus to others.

---

### TENNESSEE DENTAL ASSOCIATION.

The Ninth Annual Meeting of this Association will be held in the Senate Chamber of the Capitol, at Nashville, Commencing Wednesday, June 23, 1875, at 10 o'clock, A. M.

SUBJECTS FOR DISCUSSION.

Extraction of Teeth. Paper by Dr. S. P. Cutler, of Memphis.

Dentistry in Tennessee. Paper by Dr. W. H. Morgan, of Nashville.

Operative Dentisty. Paper by J. C. Ross, of Nashville.

Treatment of Exposed Pulp. Paper by Dr. E. S. Chisholm, of Tuscaloosa, Ala.

Treatment of Dead Teeth. Paper by R. R. Freeman, of Nashville.

Finishing Fillings. Paper by Dr. W. L. Dismukes, of Nashville.

Filling Teeth. Paper by Dr. R. Russell, of Nashville.

Mechanical Dentistry. Paper by Dr. J. H. Webber, of Springfield.

Mechanical Dentistry. Paper by Dr. D. C. Chisholm, of Franklin College.

Dental Education. Paper by Dr. S. J. Cobb, of Nashville.

Courtesy between Dentists. Paper by Dr. W. C. Sheppard, of Columbia.

Voluntary Essay. By H. E. Beach, of Clarkesville.

E. S. Chisholm, Pres. J. S. King, Sec.

---

### DENTAL SOCIETY OF THE STATE OF NEW YORK.

The Seventh Annual Meeting of this Society will be held at the Capitol, Albany, commencing Wednesday, June 30th, next, at 10 o'clock, A. M., and continue in session three days.

The Essayists for the meeting are:

C. A. Marvin, Cohesive Gold and Leaky Fillings.

O. A. Jarvis, Dental Nutrition.

Frank Abbott, Indigestion—Its causes and effects.

Frank French, Theory and Practicc.

S. B. Palmer, Success or failure in Dental Operations, Chemically considered.

S. A. Freeman, To be announced.

C. P. Fitch, To be announced.

N. W. Kingsley, To be announced.

W. H. Waite, Dentistry in England.

All members reading volunteer papers, are requested to send the subject of their essay to Dr. O. E. Hill, 160 Clinton Street, Brooklyn, who is chairman of the Business Committe.

The Essays read at the meeting will constitute the regular subjects for discussions.

It is suggested that the members and delegates come with minds well stored with "Incidents of Office Practice," such as involve points of interest, that this department of the exercises, and the discussion of the essays, may be particularly interesting and instructive.

Any member wishing to exhibit any instrument or appliances, must present them first to the Business Committee, and, if possible, before the commencement of the first day's session.

The Secretaries of the District Societies are requested to forward their reports in time for the Secretary of the State Society to make up his report.

The State Censors will convene at the Capitol, Albany, on Tuesday, June 29th, to examine candidates.

Members are requested to stay at the Delavan House, as special accommodations for our comfort have been made with the Proprietors by the Committe of Arrangements.

The meeting promises to be particularly interesting and instructive.

W. C. BARRETT, President.

CHARLES BARNES, Secretary.

### NORTHERN OHIO DENTAL ASSOCIATION.

The sixteenth annual meeting of the Northern Ohio Dental Association will be held at Put-in Bay Island, commencing on Tuesday, June 8th, 1875, at 10 o'clock, A. M. Session will continue two days, and a full attendance is desired. Members of the profession are cordially invited to be present.

E. J. WAYE, President.

C. BUFFETT, Corresponding Secretary.

ORDER OF BUSINESS.

Reading of Minutes of last Annual Meeting, Reports of Officers and Committees, reading of Essays and Discussions, Miscellaneous Business, Election of Officers—11 A. M. 2d day.

SUBJECTS FOR DISCUSSION.

Dental Caries—its Causes, What shall we do with Amalgam? Celluloid as a base for Artificial Teeth, Dental Education, Professional Etiquette, Miscellaneous.

### BIOGRAPHY.

*A Biographical sketch of Dr. T. B. Hamlin, of Nashville, Tennessee.*

Dr. T. B. Hamlin was born June 24th, 1810, at Red Hook or Hamblin's Landing, on the Hudson River, Dutchess Co., N. Y. A few months after his birth, his father moved to Windom, west of the Catskill Mountains where he died, leaving a family consisting of his wife and two sons in destitute circumstances. At the age of sixteen his mother and brother died, leaving him quite alone in the world. To be thus thrown upon his own resources so early in life, without money or education was indeed a great misfortune to the subject of this sketch; but he wisely and promptly bound himself for three years to a silver smith and watch repairer. During this time he conducted himself so well that his employer sent him to school for six months, and he also received in the latter part of his apprenticeship, some little dental instruction from a physician who was practicing medicine and dentistry together in the little village in which he lived. At the age of nineteen young Hamlin sought and obtained employment in one of the largest jewelry and watch making establishments in the city of Albany, N. Y. His industry and proficiency soon led to his promotion to the responsible position of foreman of the watch making department. In his special efforts to discharge the duties of this important position, he injured his health to such an extent that he was compelled to give up his lucrative and desirable situation and travel for his health. After traveling and resting from business for some time, his health improved and he was induced to return to Albany and secure his former position. His close confinement, however, soon told upon him and he was forced by a renewal of his disease to resign his situation, finally. From Albany he removed to Lee, Mass., where he opened a jewelry store. Shortly after and at the age of 22 years he married Miss Mary Phinney, a daughter of Auscher Phinney, a highly respectable citizen of the town of Lee. During Hamlin's stay in Lee he was attacked with rheumatism, and this in connection with his dyspepsia caused him to look to the sunny South for relief. In his travels south he went to New Orleans, where he spent one winter, deriving great benefit to his health from the change of climate. While in New Orleans he invented an instrument or tool known as the centering and pivoting tool, now in use among all watch makers and repairers. This little instrument has proved to be a very valuable invention, and if properly managed by the inventor, it might have resulted in great pecuniary profit to him. From New Orleans Hamlin moved to Wytheville, Va., where in 1835 he commenced the practice of dentistry, having studied the profession as opportunity offered ever since he received his first instructions at Windom. Soon after he commenced the practice, he joined the American Society of Dental Surgeons, and ever after was a warm advocate of dental associations, and in 1840 he recieved an honorary diploma from the Baltimore Dental College, a graceful acknowledgment on the part of that institution of his worth and professional excellence.

After ten years' practice in Wytheville and vicinity he removed to Tuscumbia, Alabama, where he practiced his profession two years.

In 1847 he moved to Nashville, Tenn., where he bought out Dr. Vancamp, paying him $6,000 for a three story brick building on Cherry Street, built expressly for a Dental office. He immediately commenced the practice at Nashville, and for 12 or 15 years did a large and lucrative business, profitable alike to himself and his patrons—to his patrons from the fact that he served them each in an honest, faithful and skillful manner so as to give them full value for every dollar they paid to him.

While Dr. Hamlin was not as extensively known in his profession as some others no one stood higher among those who knew him well, and no one knew him well, professionally or otherwise, who did not honor and respect him. Soon after he located in Nashville, he wrote and published a little book called "Importance and Care of the Teeth," and shortly after he published a small work called "Quackery Unmasked." These little unpretentious publications contained many valuable facts of much interest to the public as well as the profession, and we dare say much good was done by their general distribution. During his practice in Nashville he did a great deal in the way of interesting the people in the preservation of their teeth.

From the time he commenced the practice of dentistry, he labored with an eye single to the elevation of the dental profession, believing that to be the true way of elevating himself. The fact that he lived up to this high idea of duty resulted in his great success, in his rising from obscurity to a very high and honorable position in his profession, and also in the accumulation of a very handsome property, the mass of which was made in Nashville during the last fifteen years of his practice.

In 1861, owing to ill health he closed his professional career and after traveling four or five years through a portion of the United States and Canada and regaining his strength to some extent he returned to Nashville and purchased a farm some ten miles north of the city.

He immediately engaged with enthusiasm in the bee culture and nursery business. He soon became an accepted authority on the subject of bees, their habits, the best methods of treating them, etc. Having been more or less interested in this little creature many years before giving up his profession. He wrote a very interesting and instructive work on that subject, and received many premiums, medals and diplomas as testimonials of his accurate knowledge of bee culture. At the time of his death he owned one of the largest apiaries of the South.

Dr. Hamlin continued zealously to follow this new pursuit up to the time of his death, which was on the 24th of May 1874, in the 64th year of his age. S. J. Cobb,

Chairman of Com. on Minutes Tenn. Dental Association.

